# Symptomatic Sclerosing Pneumocytoma of Lung: A Diagnostic Challenge with Diverse Morphological Patterns

**DOI:** 10.22034/ijp.2026.2083113.3621

**Published:** 2026-08-01

**Authors:** Roshny John, Ujjawal Khurana, Tanya Sharma, Abhishek Goyal, Yogesh Kumar Niwariya, Garima Goel, Alkesh Khurana

**Affiliations:** 1 Department of Neuropathology, National Institute of Mental Health and Neurosciences, Bengaluru, India; 2 Department of Pathology and Lab Medicine, All India Institute of Medical Sciences, Bhopal, India; 3 Graphic Era Institute of Medical Sciences, Dehradun, India; 4 Department of Cardiothoracic Surgery, All India Institute of Medical Sciences, Bhopal, India; 5 Department of Pulmonary Medicine and Tuberculosis, All India Institute of Medical Sciences, Bhopal, India

**Keywords:** Sclerosing Pneumocytoma, Lung Neoplasm, Hemangioma, Diagnostic pitfall

## Abstract

**Background & Objective:**

Sclerosing pneumocytoma (SP), formerly termed sclerosing hemangioma, is a rare benign lung neoplasm often detected incidentally. Its histological diversity and dual cell population frequently complicate diagnosis, particularly in small biopsies.

**Case Presentation:**

We describe a 22-year-old female presenting with cough, right-sided chest pain, and dyspnoea for three months. Imaging revealed a well-defined, heterogeneously enhancing right middle lobe mass. Clinical and radiological impressions favored bronchial adenoma or carcinoid tumor. CT-guided biopsy suggested adenoma/adenocarcinoma due to papillary features and mild atypia. Right middle lobectomy revealed a circumscribed, tan hemorrhagic tumor. Histology demonstrated classic SP morphology with papillary, solid, sclerotic, and hemorrhagic patterns and dual surface/round cell populations. Immunohistochemistry showed nuclear TTF-1 positivity, CK7 positivity in surface cells, low Ki-67, and negative chromogranin, excluding carcinoid. No evidence of nodal or distant disease was identified, and the patient remains asymptomatic post-operatively.

**Conclusion:**

This case underscores the diagnostic challenges of SP and highlights the role of surgical resection in establishing definitive diagnosis.

## Introduction

Sclerosing pneumocytoma (SP) is a rare, benign adenoma of the lung. It presents as an asymptomatic, incidentally detected solitary pulmonary nodule. This entity is characterized by its diverse histomorphological patterns, dual population of cells, and unique immunohistochemical profile. This distinctiveness makes SP a diagnostic challenge. Herein, we present a case of SP in a young symptomatic female where the diagnosis remained concealed until surgical resection.

## Case Presentation

A 22-year-old woman presented to the Pulmonary Medicine Outpatient Department with complaints of cough, chest pain, and shortness of breath for 3 months. The patient was asymptomatic until three months ago, when she developed a dry cough that later progressed to productive cough. She had right-sided chest pain that increased with inspiration. She had dyspnea of Modified Medical Research Council (mMRC) grade II. There was no history of chronic illness, tuberculosis, or loss of weight or appetite. There was a history of weight gain and irregular menstrual cycles. Her general examination findings were unremarkable. There was no pallor, icterus, lymphadenopathy, cyanosis, clubbing, or edema. The physical examination of the chest revealed reduced breath sounds in the right infrascapular region.

Computed tomography of the chest showed a well-defined, round, smooth-walled, lobulated, heterogeneously enhancing, hypodense intraparen-chymal soft tissue mass lesion with surrounding ground-glass halo in the right middle lobe of the lung measuring 3.4 × 2.9 × 2.8 cm. A differential diagnosis of bronchial adenoma and bronchial carcinoid was offered radiologically. Positron emission tomography showed no other metabolically active lesion in the body.

A complete pathological assessment was done for this patient. Her hemogram showed hemoglobin: 13.5 g/dL, total leukocyte count: 6,770 cells/cu mm, and platelet count: 208,000/cu mm.

The patient underwent bronchoalveolar lavage (BAL). The differential counts of BAL fluid were 70% alveolar macrophages, 5% lymphocytes, 2% polymorphonuclear cells, and 23% reactive bronchial epithelial cells. The BAL was negative for malignancy, and acid-fast bacilli were not detected by Ziehl-Neelsen staining. The cartridge-based nucleic acid amplification test (CBNAAT) of the BAL was also negative.

The patient underwent CT-guided Tru-Cut biopsy. Touch imprint smears from the Tru-Cut biopsy were sent for intraoperative cytological examination. The imprint smears were cellular and showed crushing artifacts. The smears showed tumor cells arranged in acini, papillae, and also singly dispersed cells, which displayed anisonucleosis, with prominent nucleoli and abundant clear to vacuolated cytoplasm. There were many pigment-laden macrophages in the background. Considering the arrangement and nuclear features, intraoperatively, the touch imprint was diagnosed as adenocarcinoma with papillary features.

The histopathological sections from the CT-guided Tru-Cut biopsy revealed linear cores of tumor arranged in a papillary pattern with variably hyalinized fibrovascular cores and focal acinar configuration. The papillae were lined by a single layer of low columnar type of epithelium, showing clear cell change at many places. Areas showing minimal atypia in the form of anisonucleosis, multinucleation, and few prominent nucleoli were also observed. Collections of numerous hemosiderin-laden macrophages and extracellular hemosiderin were present. However, there was no increased mitosis or necrosis. Hence, a final histopathological diagnosis of papillary adenoma was given on the Tru-Cut biopsy material, and the previous diagnosis on imprint smear was amended.

**Fig. 1:**
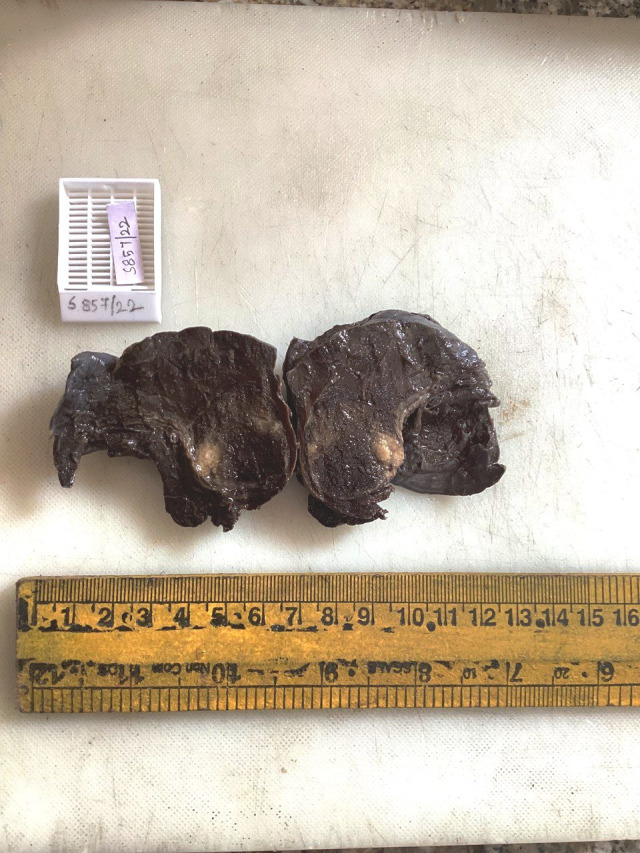
Gross photograph of cut section of partial lobectomy specimen of the right middle lobe of the lung showing a solid, tan colored, well circumscribed tumor (arrow) measuring 3cm diameter.

**Fig. 2:**
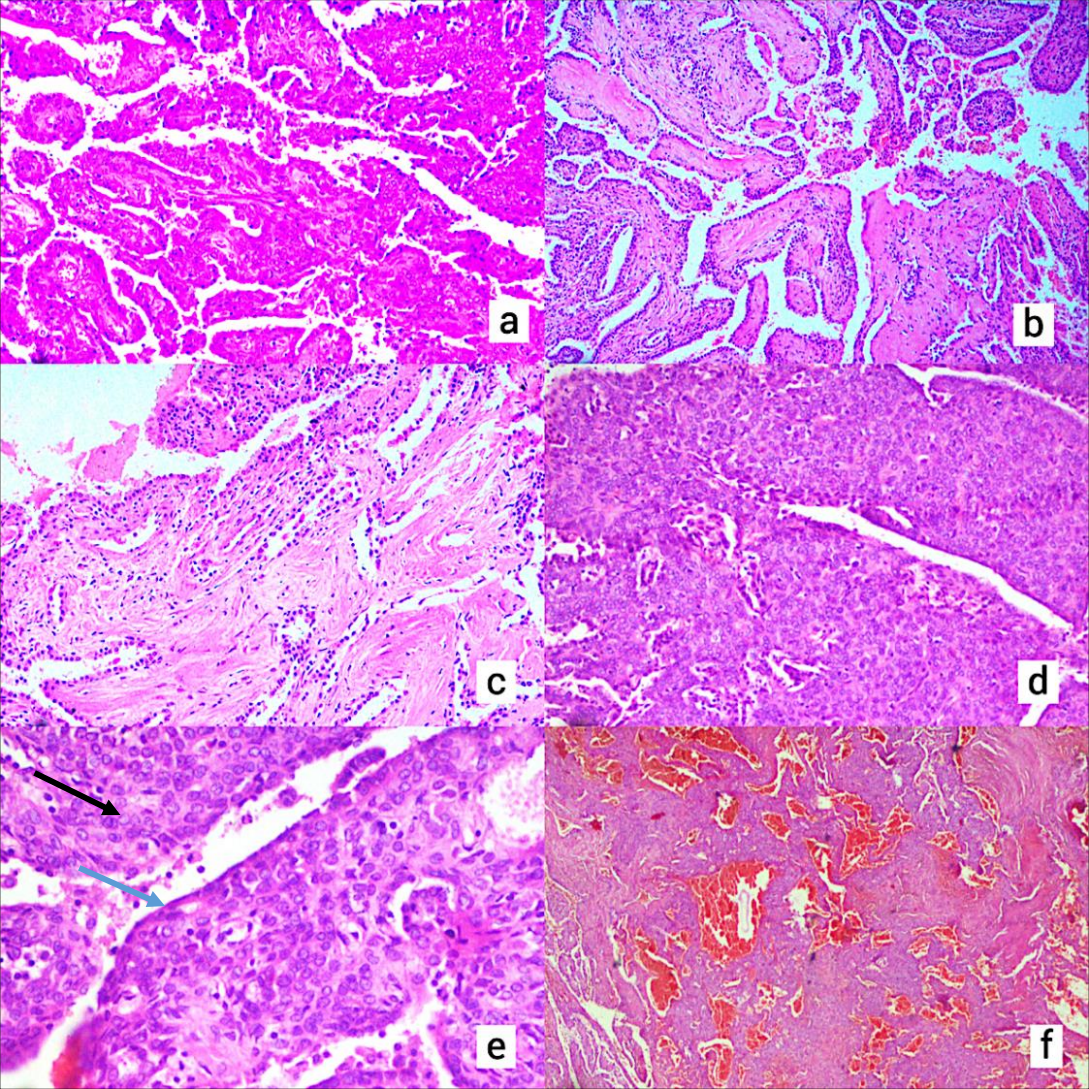
Hematoxylin & Eosin stained sections of lobectomy revealing benign pulmonary tumor displaying varying patterns including papillary [a, 10X], sclerosing [b, 10X] [c, 20X], solid [d, 10X] pattern. The solid areas showing a dual population of surface cells which are cuboidal (black arrow) and round stromal cells (blue arrow), [e, 20X] and hemorrhagic areas [f, 10X].

Three months later, the patient underwent right middle lobectomy of the right middle lobe of the lung. Gross examination of the lobectomy specimen showed a solid, tan-colored, well-circumscribed tumor with an area of hemorrhage measuring 3 cm in diameter (Fig. 1). The surgical margins, including the bronchial, vascular, parenchymal, chest wall, and pleural margins, were free of tumor on gross examination. The pleural margin was the closest to the tumor, at a distance of 1 cm. The histopathological examination of the lobectomy revealed a benign tumor showing a variety of patterns, including papillary, sclerotic, solid, and hemorrhagic patterns (Fig. 2). The papillary area showed cores of fibrovascular sclerotic stroma and epithelioid to cuboidal epithelium. There were large areas of sclerosis. The solid areas were composed of round to oval cells lined by surface cuboidal to flattened cells. There were blood-filled spaces with hemosiderin-laden macrophages. The tumor was composed of a dual population of cells comprising surface cells and round cells. Foci of multinucleation, signet ring-like cells, cells with clear cytoplasm, and calcific concretions were observed. The hilar lymph node submitted along with the specimen showed reactive lymphoid hyperplasia. There was no evidence of tumor deposits in the lymph node.

**Fig. 3:**
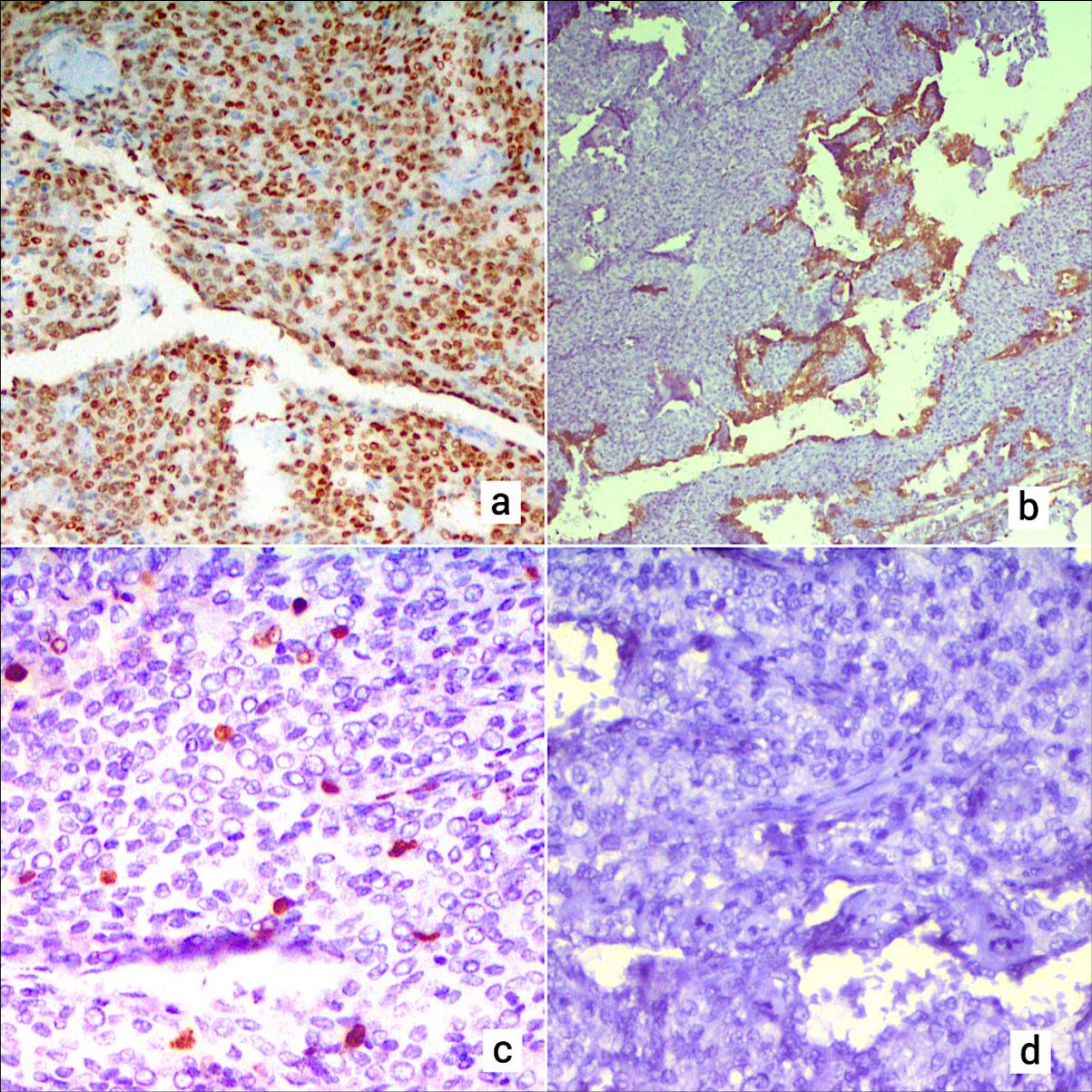
The tumour cells showed nuclear positivity for TTF-1 in both surface cells and stromal cells [a, 20X], Cytokeratin 7 (CK7) expression in surface cells, but not in stromal cells [b, 10X]. The tumour had low Ki-67 proliferative index [c, 40X] and was negative for chromogranin [d, 40X].

Immunohistochemistry showed nuclear thyroid transcription factor 1 (TTF-1)-positive surface cells and round cells. Positivity for CK7 was seen in surface cells, but the round cells were negative for CK7 (Fig. 3). The Ki-67 proliferation index was 5%. The tumor was negative for chromogranin, which ruled out bronchial carcinoid. Based on histomorphology and characteristic IHC, a diagnosis of sclerosing pneumocytoma (SP) was given on surgical resection. All resection margins were free of tumor. The patient is asymptomatic 12 months postoperatively.

## Discussion

Sclerosing pneumocytoma (SP), previously called sclerosing hemangioma of the lung, was first described by Liebow and Hubbell in 1956 in their series of seven cases(1). SP shows a slight female predominance with a wide age range of 11 to 80 years. These tumors are usually asymptomatic and are often discovered incidentally(2). Symptomatic presentations of SP are rare, although they have been reported(3). Our indexed case is a young female who had a symptomatic presentation with cough, chest pain, and shortness of breath. SP usually occurs as solitary and peripheral lesions. They have also been reported to occur in endobronchial, pleural, and mediastinal locations(4). The present case showed an intraparenchymal solitary well-defined mass lesion.

Promising advancements have occurred in the imaging diagnosis of SP. Shin et al, in their study of 76 patients with SP, described several CT characteristics, such as marginal pseudocapsule, overlying vessel, air gap, and halo sign. The mean FDG PET uptake was 1.8 (<2.5 was considered hypometabolic/benign)(5). Liu et al, in their analysis of F-18 FDG PET/CT features of 11 patients with SP, found the mean maximum standardized uptake value (SUVmax) ranged from 1.1 to 7.4. The majority of the lesions showed a moderate FDG uptake(6). Novel radiomics techniques utilize image intensity, shape, and texture to discriminate benign and malignant tumors. CT-based radiomics and textural analysis have potentiated the noninvasive imaging differentiation of SP from its differentials, such as carcinoids and lung malignancies(7,8).

SP arises from the primitive pulmonary epithelium. It is characterized by solid, papillary, sclerotic, and hemorrhagic patterns and a dual population of surface cells and round cells(2). The surface cells arise from type II pneumocytes. Yeh et al, in their comprehensive genomic and transcriptomic analysis of SP, performed RNA sequencing on 23 SPs and whole-exome sequencing on 44 SPs. Recent molecular studies suggest that activation of the PI3K/AKT/mTOR pathway may contribute to SP tumorigenesis. The authors found that AKT1 internal tandem duplications (ITDs) and point mutations were found in a large number of SPs, proving that AKT1 mutations are a major driving oncogenic event in these neoplasms. In addition, prominent SOX9 expression was seen in the round cells when compared to the surface cells(9).

SP is a benign adenoma of the lung that is often misdiagnosed as adenocarcinoma, especially in cytology samples and core biopsies(10). Rai et al reported a case of SP in a 30-year-old female who presented with hemoptysis and a right lung lower lobe mass with increased SUV uptake. The CT-guided FNAC and the biopsy were reported as adenocarcinoma. Lobectomy revealed a well-defined mass, which on histology showed the classic papillary and solid patterns with hemorrhagic areas. The dual cell population and characteristic IHC helped in clinching the diagnosis(11). David et al have reported a series of 4 cases of SP. In one of the cases, multiple CT-guided biopsies of the tumor showed morphology consistent with papillary adenocarcinoma, due to which the patient received chemotherapy. One of the CT-guided biopsies and the gross resection of the tumor showed morphology diagnostic of SP. In addition, the patient also had a coexisting typical carcinoid(12). In the present case, the intraoperative touch imprint was diagnosed as adenocarcinoma with papillary features. The low Ki-67 proliferative index helped in establishing the benign nature of the tumor.

Bronchial carcinoids are close mimickers of SP(13). Alur et al reported a case of SP that was diagnosed as carcinoid on intraoperative frozen section. The subsequent segmentectomy revealed features of SP, which was also confirmed by IHC(14). Bronchial carcinoid was a radiological differential in the present case too. The tumor was positive for TTF-1 and negative for chromogranin, thus ruling out carcinoid.

Papillary adenoma is a papillary neoplasm that consists of cytologically bland, cuboidal to columnar cells lining fibrovascular cores. Although they show a papillary pattern, the lack of dual cell population differentiates them from SP. In the present case, the Tru-Cut biopsy showed papillary arrangement of tumor; however, due to lack of high-grade features such as mitosis and necrosis, a diagnosis of papillary adenoma was favored over papillary adenocarcinoma(2).

The majority of lung tumors are malignant, and benign lung tumors are rare. When present, these benign lesions often appear as solitary pulmonary nodules. The differential diagnosis for such solitary pulmonary nodules includes a range of entities such as papillary adenoma, alveolar adenoma, bronchiolar adenoma, sclerosing pneumocytoma (SP), pulmonary hamartoma, infectious granulomas, bronchogenic carcinoma, and, in rare cases, metastatic lesions. The differentiating features of these entities are listed in the table below (Table 1).

**Table 1:** Differential features of sclerosing pneumocytoma and its mimics.

Features	Sclerosing pneumocytoma	Bronchial carcinoid	Papillary adenoma	Alveolar adenoma	Papillary adenocarcinoma
Age group	11-80 years	Typical: 45 years Atypical: 1 decade later	2 months to 70 years	39-74 years	60-70 years
Gender	F>M	F>M	M>F	F>M	M>F
Location	Peripheral (typical)	Central and peripheral	Solitary and Peripheral	Solitary and peripheral	Peripheral
Clinical features	Asymptomatic (typical) Incidental (typical)	Symptomatic due to bronchial obstruction Cough, hemoptysis, dyspnea, paraneoplastic syndromes	Asymptomatic and incidental	—	—
Gross findings	Well circumscribed Solid, variegated Grey-tan to yellow Cystic degeneration, calcification (+/-)	Well circumscribed Round to ovoid Whitish	Well circumscribed nodules White-tan to yellow	Well circumscribed, smooth, lobulated Multicystic Pale-yellow to tan	Grey white nodules with central scarring, fibrosis & pleural puckering
Microscopic findings	4 patterns: solid, papillary, sclerotic & hemorrhagic patterns 2 populations of cells: surface cells & round cells	Patterns: organoid nesting Tumor cells with fine granular chromatin	Papillae with fibrovascular core lined by single layer of cuboidal epithelium	Cystic spaces resembling alveolar spaces, lined by a single layer of type II pneumocytes overlying a stroma	Tumor cells along the fibrovascular core showing significant cytological atypia, pleomorphism, mitosis
IHC	Surface cells: TTF-1 & CK7 + Round cells: TTF-1+, CK7 - NE markers negative	NE markers+ (chromogranin, synaptophysin, etc.)	Surface cells: (not round cells) TTF-1, CK7, panCK + Low Ki-67 index	Cyst lining cells: TTF-1, napsin A + Stromal cells: TTF-1, napsin A -	TTF-1 or napsin A + High Ki-67 index

IHC: Immunohistochemistry

SP is differentiated from its mimics by its unique histomorphological patterns—papillary, sclerotic, solid, and hemorrhagic patterns—and dual population of cells comprising surface cells and round cells. The immunohistochemical expression of CK7 in the surface cells, but not in the round cells, is characteristic of this tumor. The classical morphology and IHC patterns were observed in the current case. In addition to the classical patterns, features such as multinucleation, signet ring morphology, and calcific foci were also observed in the present case.

Lymph node and distant metastasis have been occasionally reported in SP(15). In our case, the PET scan did not reveal any metabolically active lesion, and the lymph node submitted did not show any evidence of metastasis(12).

Lobectomy and sublobectomy are the preferred surgical resections of SP. The patients usually have an excellent prognosis(16). In the present case too, the patient remains asymptomatic 12 months postoperatively.

The difficulty in diagnosis of SP may be due to different reasons: (i) atypical demographic presentations such as early second decade, in contrast to typical presentations in the fifth decade; (ii) symptomatic presentations, which prioritize infections/malignancy in the list of differentials; (iii) morphological heterogeneity leading to nonrepresentative sampling from core biopsies and cytological procedures such as FNAC, TBNA, etc.; (iv) reliance on a single IHC marker such as TTF-1 alone for the diagnosis of adenocarcinoma; and (v) unfamiliarity with this tumor due to its rarity.

## Conclusions

This case report highlights the diagnostic challenges encountered in the diagnosis of SP and emphasizes the importance of surgical resection in establishing the diagnosis. SP is a rare benign lung adenoma that poses a diagnostic challenge due to its diverse histomorphological patterns. The difficulty in recognition of the dual cell type, histological heterogeneity, and dependency on the sampling type are the challenges in diagnosing this entity. Familiarity with this entity is essential to prevent misdiagnosis and mistreatment of this benign carcinoma mimicker.

## Data Availability

The data that support the findings of this study are available from the corresponding author upon reasonable request.
